# Supplementary material for: Content-rich biological network constructed by mining PubMed abstracts
Source: BMC Bioinformatics. 2004 Oct 8;5:147. doi: 10.1186/1471-2105-5-147 (PMC528731; doi:10.1186/1471-2105-5-147)
Supplement: Additional File 2 — The original results of the above study (non-essential files are deleted to keep the file size under the limit set by BMC bioinformatics). [file 1471-2105-5-147-S2.bz2 › chilibotAdditionalFile2/dip40/255ID7911228E1014/html/CDKN1A_PCNA.html]

 


 **CDKN1A** and **PCNA** 
  
Found 1649 abstracts in PubMed, retrieved 40.  
 

 What does Google say? 
 PDF only 
| .edu only 

---

**Interactive relationship** (e.g. stimulation, inhibition, etc)

**Inhibitory relationship**- p21 Waf1  [ **CDKN1A** ]  Cip1  [ **CDKN1A** ]  inhibits cyclin  [ **PCNA** ]  dependent kinase 2 activity and thereby arrests cell cycling.  Ref: 12840228 J Nutr, 2003
- Western blot analysis showed that both treatment with EGF alone and treatment with EGF after PH induce the expression of cyclins D1 and A and of p21 cip1  [ **CDKN1A** ] , but inhibites the expression of cyclin  [ **PCNA** ]  E and p27 kip1.  Ref: 12876382 Cell Physiol Biochem, 2003
- p21 Waf1  [ **CDKN1A** ]  Cip1  [ **CDKN1A** ]  exerts a key role in driving this G 2 exit both by inhibiting cyclin  [ **PCNA** ]  B1 Cdk1 and cyclin  [ **PCNA** ]  A Cdk1 2 complexes, which control G 2 M progression, and by blocking the phosphorylation of pRb family proteins.  Ref: 12881433 EMBO JEMBO J, 2003
**Neutral relationship**- p21 Cip1  [ **CDKN1A** ]  is a cyclin  [ **PCNA** ]  dependent kinase inhibitor whose abundance increases in cells exposed to radiation or other DNA damaging agents.  Ref: 12810620 Cancer Res, 2003
- Thus, PD selectively elevates p21 WAF1  [ **CDKN1A** ]  CIP1  [ **CDKN1A** ]  levels and thereby arrests the cell cycle at G1 S phase by down regulating Cyclin  [ **PCNA** ]  A Cdk2 activity.  Ref: 12819186 Carcinogenesis< MedlineTA>Carcinogenesis, 2003

**Non-interactive relationship** (e.g. studied together, co-existance, homology, etc.)

- In cell culture systems that employ human PCa cells DU145 androgen insensitive and LNCaP androgen sensitive, we found that the major polyphenolic constituent epigallocatechin 3 gallate EGCG of green tea induces 1 apoptosis, 2 cell growth inhibition, and 3 cyclin  [ **PCNA** ]  kinase inhibitor WAF 1  [ **CDKN1A** ]  p21 mediated cell cycle dysregulation.  Ref: 12840218 J Nutr, 2003
- In our study 86 tonsil cancers were analysed for HPV status by sequence analysis of polymerase chain reaction products and for the expression of cell cycle proteins p53, p21 CIP1  [ **CDKN1A** ]  WAF1  [ **CDKN1A** ] , pRb, p16 INK4A, cyclin  [ **PCNA** ]  D1 and p27 KIP1 by immunohistochemistry.  Ref: 12845651 Int J Cancer, 2003
- These events were accompanied by down regulation of the Raf 1 MEK ERK pathway as well as diminished expression of Bcr Abl and cyclin  [ **PCNA** ]  D1, cleavage of p21 CIP1  [ **CDKN1A** ]  and pRb, and induction of the stress related kinases JNK and p38 MAPK.  Ref: 12893773 BloodBlood, 2003
- Abnormalities in G1 S transition in cell cultures have been attributed to alterations in ErbB erythroblastic leukaemia viral v erb b oncogene homologue, avian signalling, cyclin  [ **PCNA** ]  D1 overexpression or disturbance of the p21 WAF1  [ **CDKN1A** ]  p21 mediated cell cycle arrest induced by p53.  Ref: 12825853 Breast Cancer Res Treat, 2003
- Immunoblot analysis revealed that BMP2 treatment caused a down regulation of p53 family members and hence of cyclin  [ **PCNA** ]  dependent kinase inhibitor p21 WAF1  [ **CDKN1A** ] .  Ref: 12850001 Biochem Biophys Res Commun, 2003
- The aim of this study was to assess the effect of Fe chelators on p53 and the mechanism involved in the chelator mediated increase in mRNA levels of the universal cyclin  [ **PCNA** ]  dependent kinase inhibitor p21 CIP1  [ **CDKN1A** ]  WAF1  [ **CDKN1A** ]  Gao, J. and Richardson, D.R.  Ref: 12869419 CarcinogenesisCarcinogenesis, 2003
- In this study, the human cyclin  [ **PCNA** ]  dependent kinase inhibitor gene p21 WAF 1  [ **CDKN1A** ]  and the murine cytokine gene granulocyte macrophage colony stimulating factor GM CSF were used simultaneously for in vivo gene therapy through systemic injection of the EGF R targeted GE7 DNA complex into murine hepatoma bearing mice.  Ref: 12833133 Cancer Gene Ther, 2003
- Most thymomas and thymic carcinomas were negative for Bcl XL, p21WAF, 1 CIP1  [ **CDKN1A** ]  and cyclin  [ **PCNA** ]  D1.  Ref: 12877732 Histopathology, 2003
- Cyclin  [ **PCNA** ]  D1 up regulation and p21 WAF1  [ **CDKN1A** ]  CIP1  [ **CDKN1A** ]  down regulation occur early in UC related carcinogenesis.  Ref: 12827612 Hum Pathol, 2003
- Expressions of  **PCNA** , p53, p21 WAF 1  [ **CDKN1A** ]  and cell proliferation in fetal esophageal epithelia Comparative study with adult esophageal lesions from subjects at high incidence area for esophageal cancer in Henan, North China.  Ref: 12854173 World J Gastroenterol, 2003
- Prediction of lymph node metastasis by p53, p21 Waf1  [ **CDKN1A** ] , and  **PCNA**  expression in esophageal cancer patients.  Ref: 12866574 J Exp Clin Cancer Res, 2003
- These latter two compounds increase the expression of p53 and its target genes such as the universal cyclin  [ **PCNA** ]  dependent kinase inhibitor, p21 CIP1  [ **CDKN1A** ]  WAF1  [ **CDKN1A** ] .  Ref: 12807743 Carcinogenesis< MedlineTA>Carcinogenesis, 2003
- These deletions define a genomic region that includes cki 1 and cki 2, adjacent genes encoding members of the Cip  [ **CDKN1A** ]  Kip family of cyclin  [ **PCNA** ]  dependent kinase inhibitors.  Ref: 12885569 Dev Biol, 2003
- These effects were accompanied by increased association of 2 CDIs, p27 kip1 and p21 waf  [ **CDKN1A** ]  cip1  [ **CDKN1A** ] , with cyclin  [ **PCNA** ]  E Cdk2 complexes.  Ref: 12866041 Int J Cancer, 2003
- The cyclin  [ **PCNA** ]  dependent kinase inhibitor p21CIP WAF  [ **CDKN1A** ]  is a positive regulator of IGF 1 induced cell proliferation in MCF 7 human breast cancer cells.  Ref: 12867429 J Biol ChemJ Biol Chem, 2003
- Inducible p53 independent regulation of the cyclin  [ **PCNA** ]  dependent kinase inhibitor p21 Waf1  [ **CDKN1A** ]  transcription is mediated through its proximal GC rich sites.  Ref: 12840224 J Nutr, 2003
- The differential regulations of cell growth, ERK activities, and cyclin  [ **PCNA** ]  D1 and p21 Cip  [ **CDKN1A** ]  WAF1  [ **CDKN1A** ]  inductions were also observed in serum enriched medium containing higher zinc concentrations.  Ref: 12816758 Am J Physiol Gastrointest Liver Physiol< MedlineTA>Am J Physiol Gastrointest Liver Physiol, 2003
- In part of the glioma cells, reduced phosphorylation of ERK 1 2 and expression of cyclin  [ **PCNA** ]  dependent kinase inhibitor 1  [ **CDKN1A** ]  or p21 was observed in co stimulation experiments.  Ref: 12825816 J Neurooncol, 2003
- METHODS Immunohistochemical avdin biotin peroxidase complex ABC method was applied to 31 cases of fetal esophageal specimens and 194 cases of adult esophageal specimens to detect the expression of p53, p21 WAF 1  [ **CDKN1A** ]  and  **PCNA**  in fetal and adult esophageal epithelia.  Ref: 12854173 World J Gastroenterol, 2003
- Phagocytosis of serum and IgG opsonized zymosan SOZ and IOZ, respectively particles into J774A.1 macrophages induced apoptosis of the cells, accompanied by the expression of p21 WAF1  [ **CDKN1A** ] , one of cyclin  [ **PCNA** ]  dependent protein kinase CDK inhibitors.  Ref: 12858021 Exp Mol Med, 2003
- Moreover, this ERK activation and cyclin  [ **PCNA** ]  D1 and p21 Cip  [ **CDKN1A** ]  WAF1  [ **CDKN1A** ]  induction were abolished by PD98059 pre treatment.  Ref: 12816758 Am J Physiol Gastrointest Liver Physiol< MedlineTA>Am J Physiol Gastrointest Liver Physiol, 2003
- Indole 3 carbinol I3C, a compound that occurs naturally in Brassica vegetables such as cabbage and broccoli, can induce a G1 cell cycle arrest of human MCF 7 breast cancer cells that is accompanied by the selective inhibition of cyclin  [ **PCNA** ]  dependent kinase 6 Cdk6 expression and stimulation of p21 Waf1  [ **CDKN1A** ]  Cip1  [ **CDKN1A** ]  gene expression.  Ref: 12840223 J Nutr, 2003
- P21WAF1 Cip1  [ **CDKN1A** ] , an inhibitor of cyclin  [ **PCNA** ]  dependent kinases, is a critical downstream effector in the P53 specific pathway of growth control.  Ref: 12840864 J Tongji Med Univ, 1999
- In conclusion, AUC shows up regulated cyclin  [ **PCNA** ]  D1 and p21 WAF1  [ **CDKN1A** ]  CIP1  [ **CDKN1A** ]  expression.  Ref: 12827612 Hum Pathol, 2003
- These data demonstrate that the additive effect is likely predominantly due to the recruitment of p27 kip1 and, to a lesser extent, p21 waf  [ **CDKN1A** ]  cip1  [ **CDKN1A** ]  into the cyclin  [ **PCNA** ]  E Cdk2 complexes.  Ref: 12866041 Int J Cancer, 2003
- Consistently with these data, Che 1 over expression inhibits proliferation of HCT116 and LoVo human colon carcinoma cell lines by activation of the cyclin  [ **PCNA** ]  dependent kinase inhibitor p21WAF1 Cip1  [ **CDKN1A** ]  in a p53 independent manner, and promoting growth arrest at the G1 phase of the cell cycle.  Ref: 12847090 J Biol Chem< MedlineTA>J Biol Chem, 2003
- In 19 of these 63 patients, p53, p21 Waf1  [ **CDKN1A** ] , and proliferating cell nuclear antigen  [ **PCNA** ]   **PCNA**  were examined immunohistologically, and to quantify the risk of lymph node metastasis, computer analysis was performed on the basis of quantification theory II, in which pathological lymph node metastasis pN was the objective variable and high or low expression of each of the three markers was the predictive variable.  Ref: 12866574 J Exp Clin Cancer Res, 2003
- Enhanced sensitivity to cis diamminedichloro platinum II of a human carcinoma cell line with mutated p53 gene by cyclin  [ **PCNA** ]  dependent kinase inhibitor p21 WAF1  [ **CDKN1A** ]  expression.  Ref: 12824923 Cancer Sci< MedlineTA>Cancer Sci, 2003
- p53 mediated induction of p21 WAF1  [ **CDKN1A** ] , a cyclin  [ **PCNA** ]  dependent protein kinase inhibitor, is known to protect cancer cells from the cytotoxic effects of anti cancer drugs or gamma irradiation.  Ref: 12824923 Cancer Sci< MedlineTA>Cancer Sci, 2003
- AIMS To correlate the expression of a series of apoptotic and oncogene markers including p53, Bcl 2, BAX, Bcl XL, p21WAF, 1 CIP1  [ **CDKN1A** ] , cyclin  [ **PCNA** ]  D1, HER 2 neu in thymic epithelial tumours with histological type, stage and resectability and to determine whether the information on HER 2 neu would be valuable in identifying patients who are eligible for anti HER 2 neu treatment.  Ref: 12877732 Histopathology, 2003
- Panaxadiol selectively inhibits Cyclin  [ **PCNA** ]  A associated Cdk2 activity by elevating p21WAF1 CIP1  [ **CDKN1A** ]  protein levels in mammalian cells.  Ref: 12819186 Carcinogenesis< MedlineTA>Carcinogenesis, 2003
- In particular, Cdk2 p21WAF1 CIP1  [ **CDKN1A** ] , an inhibitor of cyclin  [ **PCNA** ]  dependent kinase 2, was produced to a higher level and arrested the cell cycle in G1.  Ref: 12815953 Mol Biol Mosk < MedlineTA>Mol Biol Mosk , 2003
- On the other hand, prolonged ERK activity was observed with a subsequent increase of cyclin  [ **PCNA** ]  D1 and p21 Cip  [ **CDKN1A** ]  WAF1  [ **CDKN1A** ]  upon treating with 100 micro M of ZnCl2 in MSSM, and this was associated with non proliferation.  Ref: 12816758 Am J Physiol Gastrointest Liver Physiol< MedlineTA>Am J Physiol Gastrointest Liver Physiol, 2003
- p XSC and p XSeSG were significantly more effective in the upregulation of pro apoptotic genes, such as p21 CIP1  [ **CDKN1A** ]  WAF1  [ **CDKN1A** ] , p27 KIP1, APO 1, and Caspase 3, while downregulating cell growth regulatory genes, such as c myc, cyclin  [ **PCNA** ]  D1, cyclin  [ **PCNA** ]  D2 and proliferating cell nuclear antigen  [ **PCNA** ]   **PCNA** .  Ref: 12844480 Carcinogenesis< MedlineTA>Carcinogenesis, 2003
- Reduced levels of CARP 1 result in inhibition of apoptosis by CD437 or adriamycin, while increased expression of CARP 1 causes elevated levels of cyclin  [ **PCNA** ]  dependent kinase inhibitor p21 WAF1  [ **CDKN1A** ]  CIP1  [ **CDKN1A** ]  and apoptosis.  Ref: 12816952 J Biol Chem< MedlineTA>J Biol Chem, 2003
- It is unclear whether and how cyclin  [ **PCNA** ]  D1 and or p21 WAF1  [ **CDKN1A** ]  CIP1  [ **CDKN1A** ]  dysregulation contribute to ulcerative colitis UC related inflammation and colorectal carcinogenesis.  Ref: 12827612 Hum Pathol, 2003
- QUC showed cyclin  [ **PCNA** ]  D1 negative weak staining and p21 WAF1  [ **CDKN1A** ]  CIP1  [ **CDKN1A** ]  surface epithelial and upper third crypt staining expression similar to that of normal colorectum.  Ref: 12827612 Hum Pathol, 2003
- Similarly, levels of the p21 WAF1  [ **CDKN1A** ]  CIP1  [ **CDKN1A** ]  protein that is associated with the Cyclin  [ **PCNA** ]  A Cdk2 complex increased, and these increases correlated well with the down regulation of Cyclin  [ **PCNA** ]  A Cdk2 activity.  Ref: 12819186 Carcinogenesis< MedlineTA>Carcinogenesis, 2003
- Both 5% human fecal water and 4 micro mol L selenite greatly increased the mRNA level of the cyclin  [ **PCNA** ]  dependent kinase inhibitor gene p21 waf1  [ **CDKN1A** ] .  Ref: 12888658 J Nutr, 2003
- Potent iron chelators increase the mRNA levels of the universal cyclin  [ **PCNA** ]  dependent kinase inhibitor, p21CIP1 WAF1  [ **CDKN1A** ] , but paradoxically inhibit its translation a potential mechanism of cell cycle dysregulation.  Ref: 12807743 Carcinogenesis< MedlineTA>Carcinogenesis, 2003
- AIM To characterize the expression of p53, p21 WAF 1  [ **CDKN1A** ]  and proliferation cell nuclear antigen  **PCNA**  in fetal esophageal epithelia and to determine the role of these genes in proliferation of fetal and adult esophageal epithelial cells.  Ref: 12854173 World J Gastroenterol, 2003
- n = 11 were studied with cyclin  [ **PCNA** ]  D1 and p21 WAF1  [ **CDKN1A** ]  CIP1  [ **CDKN1A** ]  immunohistochemistry.  Ref: 12827612 Hum Pathol, 2003
